# An interview with Daniela Garib

**DOI:** 10.1590/2177-6709.24.1.016-026.int

**Published:** 2019

**Authors:** Daniela Garib, Carlos Flores-Mir, David Normando, Guilherme Janson, José Augusto Miguel, Steven Lindauer

**Affiliations:** 1 » Associate Professor in Orthodontics, Universidade de São Paulo, Faculdade de Odontologia de Bauru and Hospital de Reabilitação de Anomalias Craniofaciais (Bauru/SP, Brazil). » DDS, MSc and Doctorate’s degree in Orthodontics, Universidade de São Paulo, Faculdade de Odontologia de Bauru (Bauru/SP, Brazil). » Update course on Preventive and Interceptive Orthodontics, HRAC-USP (Bauru/SP, Brazil). » PhD, Harvard School of Dental Medicine (Boston/MA, USA). » Research Fellow, University of Michigan (Ann Arbor/MI, USA).; 2 » Full Professor and Orthodontic Program Director, University of Alberta Edmonton/AB, Canada). » Assistant Editor of The Angle Orthodontist, Dental Press Journal of Orthodontics and Journal of World Federation of Orthodontics. » Private practice (Edmonton/AB, Canada).; 3 » Associate Professor, Universidade Federal do Pará, Faculdade de Odontologia (Belém/PA, Brazil). » Coordinator, Specialization Program in Orthodontics, Associação Brasileira de Odontologia - Seção Pará (Belém/PA, Brazil). » Former Editor-in-Chief, Dental Press Journal of Orthodontics. » Associate editor, Progress in Orthodontics.; 4 » DDS, MSc and Doctorate’s degree in Orthodontics, Universidade de São Paulo, Faculdade de Odontologia de Bauru (Bauru/SP, Brazil). » PhD, University of Toronto, Faculty of Dentistry, Orthodontics (Toronto/ON, Canada). » Full Professor, Universidade de São Paulo, Faculdade de Odontologia de Bauru (Bauru/SP, Brasil).; 5 » Associate Professor and Coordinator, Master’s Program in Orthodontics, Universidade do Estado do Rio de Janeiro (Rio de Janeiro/RJ, Brazil). » Specialist in Orthodontics (UERJ), MSc in Pediatric Dentistry (UERJ) and Doctorate’s degree in Dentistry (UFRJ) (Rio de Janeiro/RJ, Brazil). » Diplomate of the Brazilian Board of Orthodontics.; 6 » Norborne Muir Professor and Chair, Virginia Commonwealth University (VCU), School of Dentistry, Department of Orthodontics (Richmond/VA, USA). » Bachelor’s degree (with honors), University of Pennsylvania.

**Figure:**
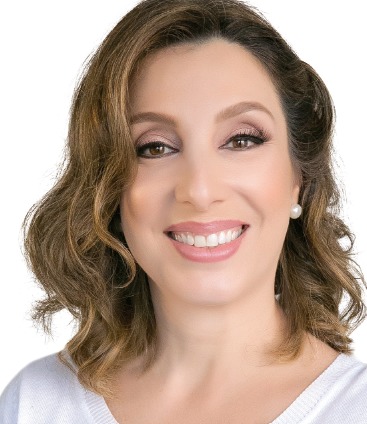

It is not hard to write about Professor Garib. Daniela is one of those self-illuminated human beings, with a light that she shares with all those who approach her. It does not dazzle us, but, faced with so many virtues, where should we begin? Daniela Garib is an Associate Professor at the *Hospital for Rehabilitation of Craniofacial Anomalies* (HRAC) and at the *Bauru Dental School* (FOB), both from the *University of São Paulo*. In Dentistry, her first steps followed her mentor, Omar Gabriel da Silva Filho, and her positive and altruistic view of life and of how to deal with people greatly resembles his views. An unbounded kindness. She got her Master’s and Doctorate from FOB-USP under the advisory of Dr. José Fernando Castanha Henriques, and completed her postdoctoral studies at Harvard School of Dental Medicine, under the mentorship of one of the legends of Orthodontics, Sheldon Peck. She has recently completed a research fellowship at the University of Michigan, working with Dr. Lucia Cevidanes. Despite her youthfulness, Daniela has already published over 160 studies in scientific journals and dozens of books and book chapters, and she has also served as an associate editor of our journal for almost a decade. An extremely competent professional, an insightful researcher with a rare intelligence, sensitive and highly educated lecturer and very clear teacher, Daniela is a wise and extremely kind professor that shares her passion with her pupils. However, it is as a mother and wife that the fascination that surrounds her is exceeded, as her daughters Laura and Helena, and her partner of a whole life, Alexandre, may tell us. Devoted, attentive and patient, Dani, as her family calls her, reflects on her work with students this simple way of bringing up her daughters. And that’s the same upbringing that she received from her parents, Léa and Isaac. In one of those encounters that only God can provide, I have had the enormous pleasure of socializing with Daniela and her beautiful family. In that context, I realized that the subtlety, an indelible feature of her personality, should never be mistaken for passivity. Daniela is assertive, authentic and resolute. A strong and principled woman. She has the perfect match of kindness and discipline, all that we want to be when we grow up! 

David Normando (interview coordinator)

Não é difícil escrever sobre a Profa. Garib... Daniela é daqueles seres humanos iluminados. Uma luz compartilhada para todos que dela se aproximam. Não ofusca, mas, diante de tantas virtudes, por onde começar? Daniela Garib Carreira é professora associada do Hospital de Reabilitação de Anomalias Craniofaciais (HRAC) e da Faculdade de Odontologia de Bauru (FOB), ambos da Universidade de São Paulo (USP). Na Ortodontia, os seus primeiros passos foram acompanhados pelo mestre Omar Gabriel da Silva Filho, com quem ela muito se assemelha na forma positiva e altruísta de enxergar a vida e lidar com as pessoas. Uma bondade incomensurável. Concluiu o mestrado e o doutorado na FOB-USP, sob orientação do Prof. José Fernando Castanha Henriques, e realizou o pós-doutorado na *Harvard School of Dental Medicine*, sob a batuta de uma lenda da Ortodontia, Sheldon Peck. Recentemente, realizou um Research Fellowship na Universidade de Michigan, junto à Profa. Lucia Cevidanes. A despeito da jovialidade, Daniela já possui mais de 160 artigos publicados em periódicos científicos, dezenas de livros ou capítulos, além de colaborar como editora-associada do DPJO há quase uma década. Profissional de extrema competência, pesquisadora perspicaz e de inteligência rara, palestrante sensível e culta, de didática límpida, Daniela é uma professora sapiente e de extrema bondade, que entrega paixão aos seus discípulos. Porém, é como mãe e esposa que ela supera o encantamento que a cerca - que nos contem Laura e Helena, suas filhas, e Alexandre, parceiro de uma vida. Zelosa, atenciosa e paciente, Dani, como é reconhecida pela família, reflete no trabalho com seus alunos a mesma forma simples de educar as suas filhas. A mesma educação que recebeu dos seus pais, Léa e Isaac. Em um desses encontros que só Deus pode nos ofertar, tenho tido o enorme prazer de um convívio pessoal com Daniela e sua linda família. Nesse espaço, percebi que a sutileza, uma marca indelével da sua personalidade, jamais deve ser confundida com passividade. Daniela é assertiva, verdadeira, determinada. Uma mulher forte e de princípios bem definidos. É o casamento perfeito entre a ternura e a disciplina, tudo o que gostaríamos de ser quando crescermos. 

David Normando (coordenador da entrevista)

**1) Some of your studies highlight that there is a genetic and hereditary basis in the etiology of dental anomalies of number and position, as well as of eruption disorders. Which correlations do you consider to be more relevant and how important are they for clinical practice? (José Augusto M. Miguel)**

**Figure 1:**
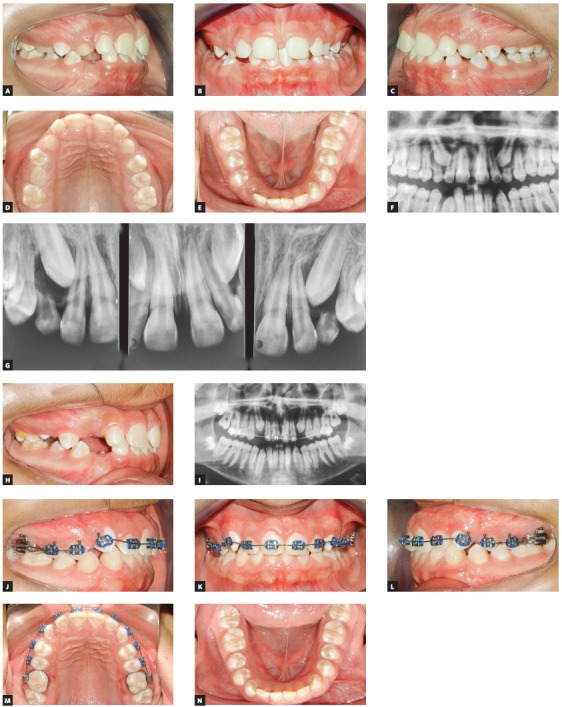
Interception of ectopic deciduous canines by extraction of deciduous canines. A-E) Baseline intraoral photos of a 10-year-old boy in the second transitional stage of mixed dentition, showing generalized enamel hypomineralization. F) Baseline panoramic radiograph shows overlapping of right maxillary canine germ and neighboring lateral incisor root, an early indicator of an ectopic tooth #13. G) Periapical radiographs obtained using the Clark’s positioning confirmed palatal position of ectopic maxillary canine crown. H) Interception was achieved by extracting right deciduous maxillary canine. I) Panoramic radiograph six months after deciduous canine extraction shows correction of eruptive path of permanent canine, now more symmetric to the left side. Due to an esthetic dissatisfaction of patient and his family, leveling using a 4 x 2 appliance was performed immediately after deciduous canine extraction, to close maxillary central interincisal diastema. J-N) Right permanent maxillary canine erupted spontaneously, simplifying corrective orthodontic treatment, whose purpose was to improve occlusion and correct positive tooth / arch-size discrepancy.

The most significant associations are those that indicate the risk of maxillary canine impaction.^1^ ^,^ ^2^ Maxillary canines are the permanent teeth, with the exception of third molars, that have the highest risk of eruption disorders. The ectopic eruption of maxillary canines, toward palatal and medial direction, results in canine impaction, which may be understood as the failure to erupt spontaneously due to unfavorable characteristics of the palate. Ectopic canine eruption considerably increases the risks for resorption of neighboring incisors. In addition, the orthodontic traction of canines is a complex treatment, with inherent unwanted consequences, such as iatrogenic resorption of neighboring teeth, with periodontal compromise and tooth discoloration. In addition, canine movement usually increases the time length of the comprehensive orthodontic treatment. The prevention of impaction is a biological benefit that should result in simpler interventions and less stress for patients, their families and for orthodontists. In this context, we highlight certain dental anomalies that indicate risk of maxillary canine impaction. Small lateral incisors, agenesis of second premolars, infraocclusion of deciduous molar and genetic hypomineralization of enamel are conditions that may be diagnosed at the early phases of occlusal development. They serve as a red flag for the occurrence of maxillary ectopic canines in the later phase of the mixed dentition. Patients with small maxillary lateral incisors have a risk of about 30% of canines taking an ectopic path. When dentists consider these risk factors, they will be better prepared to perform an early diagnosis and conduct interceptive orthodontics, which is much simpler and consists of the extraction of deciduous canines during the second transitional period of mixed dentition (Fig 1). Other interventions, such as rapid maxillary expansion and use of extraoral appliances, also have a reversing effect for ectopic maxillary canines, but have to be indicated according to the patient’s malocclusion.

**2) What are the main signs of eruption disorders of the maxillary canines? (Guilherme Janson)**

During the late mixed dentition, clinical and radiographic signs suggestive of ectopic maxillary canines may be present. The asymmetry in the position of maxillary lateral incisors during the classical ugly duckling stage and the negative palpation of the permanent maxillary canine pre-eruption bulge on the buccal side of the alveolar ridge are clinical indications of eruption disorders of the maxillary canines. A panoramic radiograph may confirm this diagnosis. The presence of overlapping images of the crown of the canine tooth germ and the lateral incisor roots, or even the roots of the central incisors, is suggestive of an ectopic eruption, most often palatally. Radiographs have a low rate of false positive results. In addition, dentists should be aware that the females, horizontal growth pattern and the presence of certain early dental anomalies, such as microdontia of lateral incisors, tooth agenesis and infraocclusion of deciduous molars, are risk factors for ectopic maxillary canines. Risk factors should increase the dentist’s attention while following up children during the mixed dentition.

**3) How early is too early to address apparent orthodontic tooth eruption problems? Does the significant push for the use of skeletal anchorage changed anything meaningfully in this specific area? Or it is simply just a change in how we apply orthodontic forces to erupt teeth? (Carlos Flores-Mir)**

The mixed dentition is the ideal timing for the interception of eruption disorders because eruption problems are revealed during the phase when permanent teeth take the place of deciduous teeth. The resolution of eruption disorders has been minimally affected by the advances in skeletal anchorage techniques. Skeletal anchorage has contributed remarkably to our capacity to perform tooth intrusion procedures, to close edentulous spaces and to perform the orthopedic treatment of Class III malocclusion. The longitudinal follow-up of dentition development and our sharp eyes to diagnose early signs of eruption disorders seem to be essential for their effective management.

**4) What is the main advantage of the expander with differential opening, over the conventional Haas or Hyrax expander? (Guilherme Janson)**

**Figure 2:**
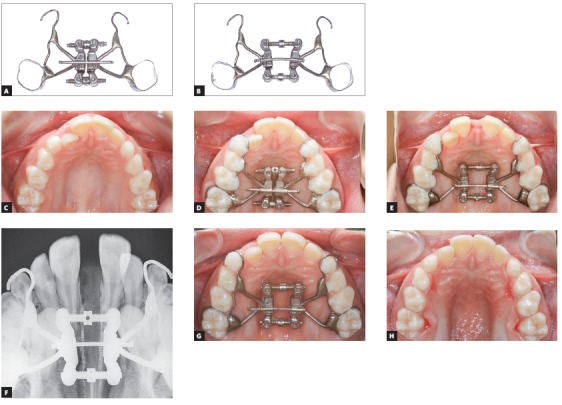
Differential Maxillary Expander. A, B) Trapezoid design, with the screw arms diverging anteriorly after different amounts of opening of anterior and posterior screws. C-H) Clinical sequence of rapid maxillary expansion using Differential Maxillary Expander in nine-year-old female patient with no cleft lip and palate. The anterior screw was activated 8 mm while the posterior screw was activated 5mm.

The basic difference lies on the design of the expansion screw. In conventional expanders, such as Haas and Hyrax, screw activation produces parallel expansion, as the palatal bars move apart at a similar rate in the anterior and posterior regions of the dental arch. Therefore, increases of intermolar width are expected to be similar to intercanine width increases. In contrast, the differential expander has a trapezoid screw opening design with the lateral bars diverging toward anterior. In this case, expansion in the canine region is greater than in the molar region (Fig 2). The Differential Expander, therefore, is an alternative to conventional expanders when the orthodontist plans to increase the anterior expansion. This expander was first designed for patients with bilateral complete cleft lip and palate, in whom the transversal deficiency is greater in the canine than in the molar region. For these patients, we used to start with a conventional Haas or Hyrax expander followed by a fan-type expander. Currently, we do the same using a single appliance, the expander with differential opening. Its advantage is the reduction of treatment time and number of appointments, thus reducing the burden of care. Patients without clefts may also be eligible to treatment with this type of expander when their maxillary deficiency is more severe in the anterior region of the dental arch. Moreover, relapse after maxillary expansion is greater in the intercanine distance compared to the intermolar distance. In this case, the Differential Expander may be indicated to individualize overcorrection in the regions of greater relapse.

**5) The beginning of this century was marked by scientific studies that criticized the efficiency of treatment during mixed dentition or in two phases. Of the protocols used in your clinical practice, which you still consider to be advantageous to patients and which have been altered because of new scientific evidence? (José Augusto M. Miguel)**

Efficiency is an important goal to pursue in Orthodontics, but not the only one when selecting a treatment plan. Benefits such as the anticipation of smile esthetics, self-esteem and well-being, as well as the establishment of an adequate functional occlusion, the possible simplification of the second phase (comprehensive orthodontic treatment) and the limitation of iatrogenic problems associated with root resorption, bone dehiscence and white spots, should be weighed in order to perform an adequate decision regarding treatment timing. When benefits are greater than the cost of less efficiency, the treatment in two phases may be recommended. The treatment stability performed in one and two phases should also be taken into consideration. When treating an open bite, for example, the chances of relapse in cases of early interventions and corrective interventions are quite different. Therefore, we believe the treatment in two phases is positive in cases of crossbite, maxillary and mandibular width deficiency, crossbites and Class III malocclusion. A recent study revealed that interceptive orthodontics might correct up to 75% of the complexity of Class III malocclusions, thus reducing mechanical efforts of comprehensive orthodontic treatment. Eruption disorders, such as ectopias and transpositions, are also treated using simpler methods of treatment in the pre-adolescent phase. Crowding of permanent incisors may be corrected at an early stage, when the child is unhappy with his self-image. In cases of crowding, the decision to treat in one or two phases should be centered on patients and their families.

**6) What are the clinical implications of your research about aging of the occlusion and its impact on dentofacial esthetics? I mean, what should our occlusal and esthetics target be when orthodontically treating our patients? Should we change our current orthodontic goals? (Carlos Flores-Mir)**

We should avoid recommending clinical crown lengthening in very young patients. Apical migration of the gingival margin is significant from the beginning of adolescence to 18 years of age, and continues along the whole life. When we complete a comprehensive orthodontic treatment of a 13-year-old adolescent and find that the clinical crown of the maxillary incisors are short in relation to the mesiodistal diameter, we should wait until the patient is an adult to recommend clinical crown lengthening. Crown height will definitely increase significantly in the five years to come. When crown height is markedly discrepant, crown lengthening should be conservative and adequate to the patient’s age. Extensive surgical increases should be avoided, because they may add characteristics of early aging to young patients. Another recommendation is to augment worn incisal edges or the incisors and canines along adult life. Finally, the orthodontist should avoid the excessive use of intrusive movements of maxillary incisors in young patients, so that the smile is not prematurely aged. In adult patients, extrusion of maxillary incisors may be an antidote to smile aging.

**7) Is there any difference in facial maturation between sexes? (Guilherme Janson)**

As in growth, sexual dimorphism is clear in maturation and aging. This conclusion is based on the follow-up of individuals with normal occlusion for 40 years, which we had the pleasure to conduct in Bauru, Brazil, as well as in classical studies in the literature, such as the doctorate thesis by Behrents, who longitudinally analyzed nontreated individuals from North American growth centers. Male faces tend to become straight in maturity. In women, the face becomes more convex. These differences are associated with mandibular growth in adulthood. In men, the mandible grows and rotates upward because of a greater elongation of the mandibular ramus. At the same time, mandibular incisors move lingually and overjet may be reduced. In women, the mandible rotates downward and reduces menton protrusion. Incisors tend to move labially as compensation. One of our recent studies showed that, in addition, the decrease in the thickness of the upper and lower lips during maturity is greater in men than in women. In contrast, soft-chin thickness increases more in men than in women, which contributes to the reduction of face convexity in men during aging.

**8) Your research group recently published some fantastic studies about dentition maturation. What are the most important learning from these studies? (David Normando)**

**Figure 3:**
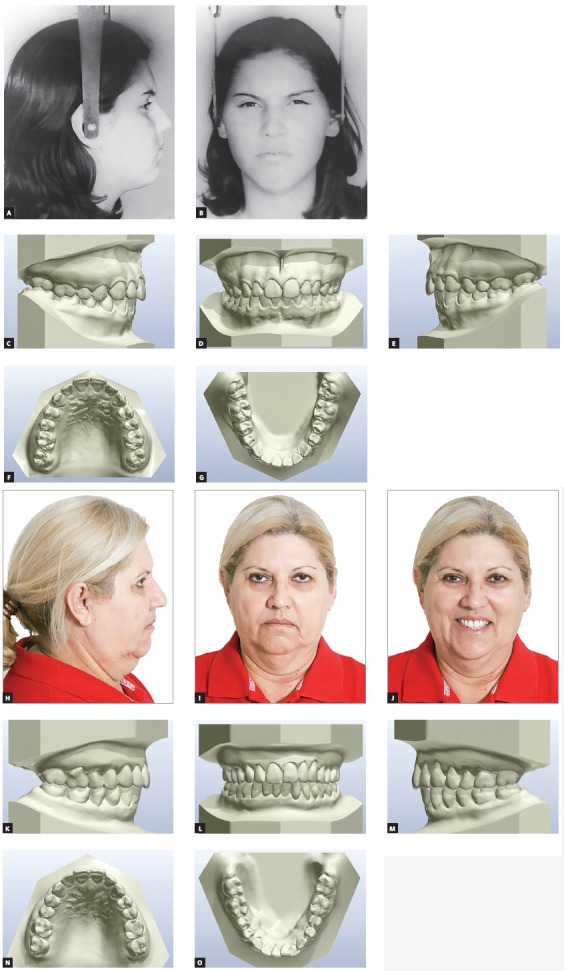
Normal occlusion maturation. A-G) Thirteen-year-old female patient with complete permanent dentition and untreated normal occlusion (continues on the next page).

The Department of Orthodontics of FOB-USP, which has an important sample of White-Brazilian with normal occlusion, provided us with a unique opportunity. The Department collected records of individuals at ages of 13 and 17 years in the 1960’s and 70’s. I wanted to give my contribution to the faculty team. Three years ago, together with my graduate students, we started recruiting these same individuals at the age of 60 to study aging of the normal occlusion. It has been an exciting challenge to find them four decades later. We learned that normal occlusion, despite some mild changes due to the increase of clinical crowns, decrease of maxillary and mandibular arch perimeter, and increase of irregularities in mandibular incisors (Fig 3), is the most stable feature of the face during the aging process. The face changes substantially: Men lose hair, the skin changes its texture, the lines and folds deepen, the nose and the ears grow, the zygomatic process loses a lot of volume, menton gains volume, the upper lip becomes thinner and move downward. During this study, it became very clear that the smile is the most notable memory of youth. In individuals with satisfactory dental care, the smile is the most concrete trait of youth to remain along time. As professionals, we should promote the preservation of a young smile by reconstructing worn incisal edges, whitening discolored teeth and avoiding excesses in the indication and magnitude of clinical crown lengthening. In young patients, intrusion of maxillary incisors should be avoided whenever possible, unless the patient has an excessive exposure of the clinical crown at rest. In adults, the extrusion of maxillary incisors combined with intrusion of mandibular incisors may be appropriate to achieve a young appearance.

**9) Do you think there has been sufficient focus on research regarding cleft lip and palate in the orthodontic literature? (Steven Lindauer)**

The prevalence of cleft lip and palate is high, and craniofacial anomalies are one of the main concerns of the World Health Organization, but very little attention to orofacial clefts is found in the orthodontic literature. A classical example is the rapid maxillary expansion (RME), which may be one of the most investigated procedures in Orthodontics. However, when we started to study RME in cleft lip and palate (CLP), exactly ten years ago, evidence was scarce. A possible explanation is that CLP rehabilitation is usually conducted in specialized centers whose main purpose is to provide care, rather than education and research mission. Another probable explanation is that CLP requires longitudinal studies. The impact of primary plastic surgery and early interventions is only understood after longitudinal follow-up (5 to 18 years) of the development of children. This may be the primary obstacle for accomplishing research. At the same time, the lack of evidence of care is the reason why the burden of care and orthodontic overtreatment are still very large in CLP. When the cost-benefit ratio of the different interventions is unknown, professionals tend to overtreat unnecessarily and without any substantial long-term impact. Studies should aim at elucidating efficient and effective treatments whose results are stable in the long term.

**10) What advice would you give to a young researcher who was interested in working in the field of cleft lip and palate? (Steven Lindauer)**

The first piece of advice I would give to a young researcher interested in the field of craniofacial anomalies would be to read all the manual *“Global strategies to reduce the health-care burden of craniofacial anomalies”* (WHO, Geneva, 2002)^3^ and, immediately after, the Eurocleft, Americleft and Scancleft intercenter studies^4^ ^-^ ^10^. This would be the scientific “baptism” for future researchers in the field of CLP research. Many questions not yet answered will clearly highlight during these readings. Finally, I would recommend special attention to study designs centered on the patient.

**11) The HRAC-USP does a wonderful job in handling CLP patients longitudinally. It is a world renowned centre in this regard. What are the five key concepts that an orthodontist that is not related to a hospital or orthodontic program need to know about CLP management? (Carlos Flores-Mir)**

1. Dedicate to keep long-term records of your CLP cases.
2. Discuss treatment planning using an interdisciplinary approach. Learn to recognize the limitations of your specialty and listen to professionals of other areas.
3. Avoid overtreatment. Make simpler treatment plans. Focus only on orthodontic procedures that have a long-term impact.
4. Invest your time talking to patients and their parents or guardians. Take their desires and opinions into consideration when planning your cases. Planning may be individualized according to the expectations of each family, which may vary broadly.
5. Aim at efficiency incessantly. If you have to choose between two appliances, choose the most efficient. Try to contribute to the reduction of the burden of care, which is substantially high for patients with CLP.

**12) What changes have you seen recently that have improved treatment outcomes for cleft lip and palate patients? Is there anything new that you see coming soon? (Steven Lindauer)**

Bone-anchored maxillary protraction (BAMP) with miniplates opened a new perspective of orthopedic management for patients with CLP, successfully treating moderate and severe maxillary deficiency at an earlier stage and less invasively than orthognathic surgery. As rehabilitation professionals, we get comfort from the fact that we give the patient with CLP the opportunity to go through adolescence with a better facial profile, a factor that is especially important for his psychosocial health. We still have to clarify the degree of stability of this protocol in the long term, and learn to predict more accurately the treatment prognosis, in order to identify the patients that best respond to it. We have no doubt that patient and family discipline is essential for treatment success. Skeletal age and facial pattern probably affect orthopedic results and should, thus, be better investigated. When we learn to identify unfavorable features for BAMP therapy, we will be prepared to suggest early orthognathic surgery before skeletal maturity. This is the focus of one of the current studies in our rehabilitation center, and we expect to share some evidence in the near future. Another relatively new procedure in the care of patients with CLP is presurgical orthopedics with nasoalveolar molding. HRAC-USP has decided to wait for longitudinal evidence of the cost-benefit of this intervention before including it in the rehabilitation protocol. Finally, great advances in tissue engineering may benefit patients with CLP in the future, first improving alveolar bone reconstruction procedures and, later, the use of soft tissues in the reconstruction of lip and palate. 

**13) The HRAC-USP, your rehabilitation center, has had a relevant participation in providing quality scientific evidence about patients with CLP. There has been a growing trend toward the use of absolute anchorage to improve the response to orthopedic treatment. How can this affect planning and prognosis for these patients? (José Augusto M. Miguel)**

**Figure 4:**
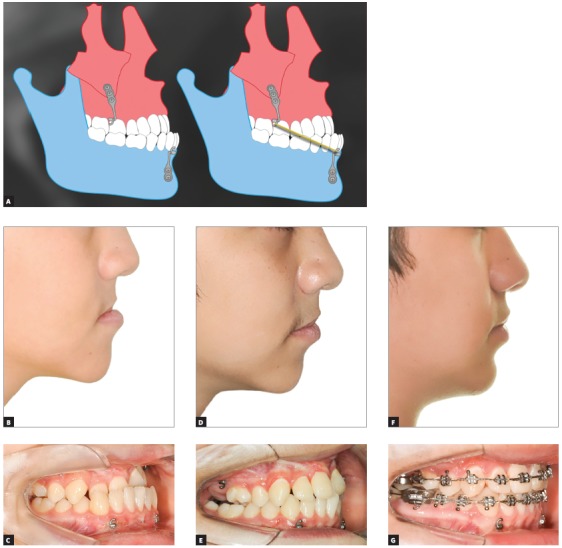
Bone-anchored maxillary protraction (BAMP) with miniplates in patient with complete and unilateral cleft lip and palate. A) Illustration of miniplate positioning in posterior region of maxilla and anterior region of mandible, interconnected with Class III elastics (250g force). B-G) Patient with complete and unilateral cleft lip and palate at initial orthopedic intervention at 12.5 years of age: B, C) initial photographs, D, E) 12 months after BAMP with miniplates and F, G) after completion of corrective mechanics.

Bone-anchored maxillary protraction (BAMP) with miniplates brought new treatment perspectives for patients with CLP. Approximately 60% of the patients with unilateral complete CLP have some sagittal maxillary deficiency due to the restrictive effect of plastic surgeries, lip and palate repair in early childhood. When maxillary retrusion is moderate or severe, prognosis is poor because of the need of orthognathic surgery to advance the maxilla at the end of facial growth. The greatest limitation of orthognathic surgery is the timing to perform it. Patients have to go through the critical phase of adolescence with the facial deformity, waiting for skeletal maturity before undergoing the surgical maxillary advancement. In contrast, BAMP therapy with miniplates is performed at the late mixed dentition or at the early permanent dentition. Therefore, facial and occlusion deformities are corrected at an earlier age. Cooperative patients with disciplined families respond very well to the treatment using full-time Class III elastics. Another important advantage of BAMP is the fact that active retention may be kept until the completion of facial growth, improving the prognosis of stability and preventing orthognathic surgery (Fig 4). A discriminatory analysis of patient adherence to the use of Class III elastics should be the next step to determine the prognosis of this new type of treatment.

**14) You studied with Professor Omar Gabriel da Silva Filho and other great names in Brazilian and global Orthodontics. What mark did they leave in your academic, clinical and personal life? (David Normando)**

We carry the influence of mentors that we admire greatly. We are grateful to all the masters that generously dedicated their lives to sharing what they learned through their reading efforts, experience and clinical perceptions, as well as through deep reflections and critical thinking. An important lesson that I have learned from my professors was that teaching is not enough. A professor should inspire. We learn more when we are emotionally touched. The great Greek philosophers and the brilliant Albert Einstein went beyond the limits of the classroom. They walked together with their students to admire nature, as they instigated them with their infinite inquiries. Teachers have to be at the same time passionate and discerning. Passion for science and discernment in the search for truth should be inseparable. As soon as I graduated in Dentistry, my first Orthodontic training was a two-year residency in HRAC-USP, under the supervision of Dr. Omar Gabriel da Silva Filho. His dynamic lectures enchanted us. Soon I started the Master’s Degree program at FOB-USP, and our professors taught us to search for information on our own. We gave dozens of seminars every week under their very rigorous academic scrutiny - and here I express my gratitude to the FOB-USP faculty members, who today are my department colleagues. When I was finishing my Master’s, I had a meeting with Dr. Silva Filho in his office and had the opportunity of thanking him. Those two years of self-study during my Master’s, digging through the literature, showed me that my mentor had been rigorously truthful to both science and evidence in his lectures: truth was tied to all the magic and poetry of his speech. My professors also taught me the value of constant clinical experience as an essential part of being an professor in Orthodontics. I experienced the work in a private clinic for 13 years, and now, despite my full-time commitment to teaching and research, I have not abandoned clinical practice. I see my own patients at the University, an activity that I believe to be essential to the advancement of teaching. My international post-doctorate fellowship supervisors taught me the value of balance in our lives. A teacher may and should have other interests and expertise. Life is too broad and long to be dedicated to a single task. Dr. Sheldon Peck was an admirable researcher and clinical orthodontist and, at the same time, an art connoisseur and collector. He collected Rembrandt’s drawing and his art lectures were as outstanding as his classic orthodontic lectures. Human beings are multifaceted.
